# Perilimbal sclera mechanical properties: Impact on intraocular pressure in porcine eyes

**DOI:** 10.1371/journal.pone.0195882

**Published:** 2018-05-02

**Authors:** Xiaofei Man, Elizabeth Arroyo, Martha Dunbar, David M. Reed, Neil Shah, Larry Kagemann, Wonsuk Kim, Sayoko E. Moroi, Alan Argento

**Affiliations:** 1 Department of Ophthalmology and Visual Sciences, University of Michigan, Ann Arbor, Michigan, United States of America; 2 Department of Ophthalmology, Xin Hua Hospital Affiliated to Shanghai Jiao Tong University School of Medicine, Shanghai, People’s Republic of China; 3 Department of Mechanical Engineering, University of Michigan-Dearborn, Dearborn, Michigan, United States of America; 4 Department of Mechanical Engineering, Northwestern University, Evanston, Illinois, United States of America; 5 Department of Ophthalmology, Loyola University Medical Center, Maywood, Illinois, United States of America; 6 Department of Ophthalmology, NYU Langone Medical Center, NYU School of Medicine, New York, New York, United States of America; 7 Division of Ophthalmic and Ear, Nose and Throat Devices, Office of Device Evaluation, Center for Devices and Radiological Health, Food and Drug Administration, Silver Spring, Maryland, United States of America; Bascom Palmer Eye Institute, UNITED STATES

## Abstract

There is extensive knowledge on the relationship of posterior scleral biomechanics and intraocular pressure (IOP) load on glaucomatous optic neuropathy; however, the role for biomechanical influence of the perilimbal scleral tissue on the aqueous humor drainage pathway, including the distal venous outflow system, and IOP regulation is not fully understood. The purpose of this work is to study the outflow characteristics of perfused porcine eyes relative to the biomechanical properties of the perilimbal sclera, the posterior sclera and the cornea. Enucleated porcine eyes from eleven different animals were perfused with surrogate aqueous at two fixed flow rates while monitoring their IOP. After perfusion, mechanical stress-strain and relaxation tests were conducted on specimens of perilimbal sclera, posterior sclera, and cornea from the same perfused eyes. Statistical analysis of the data demonstrated a strong correlation between increased tangent modulus of the perilimbal sclera tissues and increased perfusion IOP (R^2^ = 0.74, p = 0.0006 at lower flow rate and R^2^ = 0.71, p = 0.0011 at higher flow rate). In contrast, there were no significant correlations between IOP and the tangent modulus of the other tissues (Posterior sclera: R^2^ = 0.17 at lower flow rate and R^2^ = 0.30 at higher flow rate; cornea: R^2^ = 0.02 at lower flow rate and R^2^<0.01 at higher flow rate) nor the viscoelastic properties of any tissue (R^2^ ≤ 0.08 in all cases). Additionally, the correlation occurred for IOP and not net outflow facility (R^2^ ≤ 0.12 in all cases). These results provide new evidence that IOP in perfused porcine eyes is strongly influenced by the tangent modulus, sometimes called the tissue stiffness, of the most anterior portion of the sclera, i.e. the limbus.

## Introduction

Glaucoma remains a global burden of eye disease with a projected increase from 70–90 million individuals worldwide affected with glaucoma to 111 million individuals by the year 2040 [1]. In the US, the National Eye Institute estimated 3 million people, who are 40 years or older affected by primary open-angle glaucoma (POAG) in 2010, and this is projected to increase to 4.3 million in 2030 and to 6.3 million in 2050 [2]. Older age and relative elevated intraocular pressure (IOP) are the major risk factors for glaucoma, and the main treatment approach for glaucoma targeted effective IOP reduction [3]. Earlier studies focused on the trabecular meshwork and juxtacanalicular tissues to understand mechanisms that underlie elevated IOP [4]. In a classic study on enucleated human eyes, Grant concluded that the major site of outflow resistance is within the trabecular meshwork [5]. A follow-up study with perfusion at normal IOP demonstrated that 49% of outflow resistance was eliminated by complete trabeculotomy, and at higher IOP, 71% of outflow resistance could be eliminated [6]. However, the mechanisms that contribute to the remaining outflow resistance distal to Schlemm’s canal has more recently been appreciated in the aqueous veins and intrascleral veins that are within the perilimbal sclera [7].

Early *in-vivo* evidence for the role of these aqueous veins on IOP regulation was demonstrated in [8] where topical epinephrine lowered IOP by a combined mechanism of reducing blood flow and increasing aqueous flow into the veins. The observed IOP reduction is likely not due to decreased production of aqueous humor in the ciliary body because local application of epinephrine to the ciliary region increased aqueous humor production. Rather, this pharmacological effect on the complex IOP dynamics in this model may be attributed to increased aqueous outflow through the veins. Using detailed histology and newer imaging techniques, the regional variation of aqueous humor outflow through the trabecular meshwork into Schlemm’s canal [9], the collector channels [10], and aqueous veins [10] is established in human eyes.

As the sclera constantly bears the load of IOP, its biomechanical stress can influence areas where either nerves or blood vessels penetrate the sclera. While there is extensive knowledge on the relationship of the posterior scleral biomechanics and IOP load on glaucomatous optic neuropathy [11, 12], the role for biomechanical influence of the perilimbal scleral tissue on the aqueous humor drainage pathway, including the distal venous outflow system, and IOP regulation is not fully understood. The purpose of this study is to integrate biomechanical studies on the eye’s cornea-perilimbal scleral tissues with aqueous humor dynamic perfusion studies. The present work is novel in that the outflow characteristics of perfused eyes is studied relative to the biomechanical properties of the perilimbal sclera, the posterior sclera and the cornea tissues. In particular, experiments were designed to determine if the biomechanical properties of the perilimbal scleral tissues, in which the aqueous veins reside, influence net outflow facility and IOP.

## Materials and methods

### Porcine eye preparation

Intact porcine eyes were obtained from two local slaughterhouses: Milligan’s Northwest Meat Market, 7051 Standish Rd., Jackson, Michigan, USA, and Scholl Slaughterhouse, 1305 S. Piotter Hwy., Blissfield, Michigan, USA. Both have been inspected and approved by the United States Department of Agriculture, Food and Safety Inspection Services for humane handling of livestock. Eyes were kept in closed plastic bags in a small, closed container at 4°C with all fat intact to maintain hydration until preparation for testing. Perfusion tests were conducted on the same day the animal was slaughtered. Preparation and handling of samples followed the procedure established previously [13]. All fat, extraocular muscles and peribulbar tissues were removed. The optic nerve was trimmed flush to the scleral surface. Eyes were kept hydrated during preparation by periodically moistening with balanced salt solution (BSS). Before perfusion testing, each eye was gradually warmed to 37°C by inserting it in a warmed hydration tube containing gauze moistened with BSS. This maintains humidity in the vials at about 95%, as monitored by a humidity probe inserted in the vial [13, 14].

**Fig 1:**
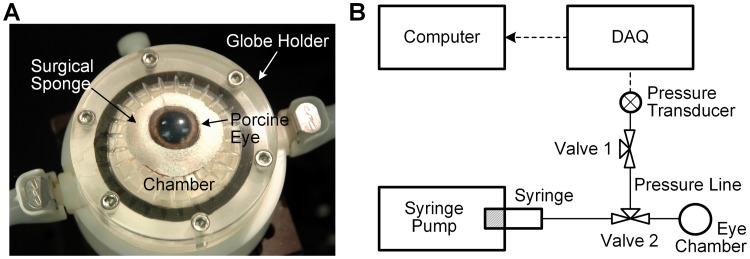
Perfusion test. (A) Tests occurred in a water jacketed eye chamber consisting of a water jacketed holder and cover (cover not shown for clarity). Here a porcine eye is shown resting in the holder during the 15 minute equilibrium period while a BSS moistened hydration sponge is applied to the eye and environmental conditions equilibrate in the chamber. (B) Perfusion system set-up. The perfusion syringe is set in a syringe pump and connected to a tube with a 3-way valve (Valve 2) which connects to a pressure transducer and a needle inserted in the posterior chamber of the eye that sits in the eye-holder. Valve 1 facilitates the removal of bubbles from the system during set-up. Pressure signals are recorded via the data acquisition system and recorded by the computer.

After this preparation, each eye was placed in a custom built, water-jacketed globe holder (Fig 1A) for pre-hydration, pre-conditioning and the perfusion test. The holder is fitted with a cylindrical plastic cap having 67 mm diameter and 105 mm height (not shown). The system (Digital One, NESLAB RTE10, Thermo Electron, Newington, NH) circulates water heated to 37°C in a closed chamber that encircles the posterior two-thirds of the eye. This serves the function of warming the posterior portion of the eye without allowing water to contact the eye. Tissue wipes moistened with BSS were placed under the eye to maintain hydration, raise it slightly off the bottom surface and permit minor adjustments to eye position. No glue or fixation was used since these produce stress and strain focally near the attachment points. This support system allows the eye to freely expand during pressure changes. A ring shaped Weck-Cel^**®**^ surgical sponge trimmed and moistened with BSS was placed over the perilimbal sclera, as seen in Fig 1A, for 15 minutes to maintain its hydration as the humidity and temperature equilibrated in the small chamber. During perfusion testing, the water jacket and plastic cap maintain a humid environment for the test and regulate the posterior two-thirds of the eye at physiologic temperature while the anterior segment of the eye is open to a damp, slightly cooler environment.

### Whole globe perfusion

The experimental perfusion set-up is shown in Fig 1B. First, the tubing (Tygon ND-100-65, ADF0004, 5/32 inch outer, 3/32 inch inner, Saint-Gobain North America, Malvern, PA), 21 gauge needle and valve were assembled and all air bubbles removed by submerging the assembled parts in BSS warmed to 37°C and drawing the fluid in and out of the system. The assembled system, loaded with BSS, was then placed in position for testing. A pressure sensor (Argon DTXPlus, Argon Critical Care Systems Singapore Pte. Ltd, Singapore) was connected to the valve and air removed from it by using the syringe to force BSS through it and out its relief port. During tests, pressure sensor output was continuously acquired using a data acquisition system (PowerLab 8/35, ADInstruments, Bella Vista, Australia).

A fresh 21 gauge needle was used to enter the anterior chamber through the peripheral clear cornea with horizontal advancement that was parallel to the iris. The lens was not violated and the needle tip was advanced to just posterior to the iris margin, reaching the posterior chamber of the eye. The globe holder and apparatus was arranged to support a stable horizontal position of the needle using lab tape on the tubing material. This support system on the tubing line to the needle prevents its weight from loading the cornea.

Since enucleated eyes lose pressure, the globes were pre-conditioned using the following tissue conditioning protocol to restore them close to a physiologic state. The globe’s IOP was gradually increased to 15 mmHg over a two-minute period and then held constant for two minutes. The valve between the globe and the syringe was then closed allowing the tissue to relax and the globe’s IOP to accordingly decrease. After three minutes of relaxation, the cycle was repeated until the relaxation phase profiles stabilized, indicating the eye established a reference state and the tests are repeatable. All eyes achieved a stable relaxation phase on the third cycle. This pre-conditioning process does not load the eye beyond its normal physiological range in its intact state, so there was no concern about unnaturally loading the eye.

Constant flow rate perfusion was conducted in these experiments using fixed flow rates of 3 and 6 μl/min. Immediately following pre-conditioning, the syringe pump (New Era NE-100, New Era Pump Systems, Inc., Farmingdale, NY) was set to a flow rate of u_1_ = 3 μl/min and the IOP monitored and recorded continuously. When the IOP reached a steady IOP value P_1_, the flow rate was changed to u_2_ = 6 μl/min until a second steady IOP value P_2_ was reached. Steady state IOP was defined by the condition that the change in IOP after a 15 minute period divided by the IOP at the start of the period be less than 0.01. Net outflow facility, C, was calculated using the following definition from [15].

$$\text{C}=({\text{u}}_{2}-{\text{u}}_{1})/({\text{P}}_{2}-{\text{P}}_{1})$$ (1)

### Specimen preparation and biomechanical testing

Stress-strain and relaxation tests were conducted on perilimbal sclera, posterior sclera and cornea tissue specimens dissected in a standardized approach from the perfused eyes using an established method [13]. During dissection, tissues were periodically moistened with BSS to maintain hydration. After dissection, specimens were placed in 50 ml polypropylene tubes containing BSS moistened wipes that were then placed in 4°C coolers. Before test, specimens were warmed to 37°C in the hydration tubes. For the perilimbal tissues, two specimens were dissected from the perilimbal sclera superior and inferior and immediately adjacent to the cornea using a custom-made, dog-bone shaped cutting die (TestResources, Shakopee, MN). In [16], the use of dog-bone shaped samples rather than rectangular strips is recommended for tensile tests of porcine sclera to minimize stress concentration near testing grips. Extraction was made by carefully cutting the eye in half along the coronal plane at the equator. The internal underlying tissues of the ciliary body, lens, iris and any peripheral retina and choroid were removed. Four small radial incisions were made to the anterior segment cup to lay this tissue flat on a wax dissection tray. The die was pressed through the superior and inferior perilimbal tissues with the axis of the die directed in the nasal-temporal direction. One sample was randomly selected for a stress-strain test and the other for a relaxation test. Specimen thickness, averaged using measurements at two or three locations on a sample, was recorded for each sample to calculate engineering stress. Because of the orientation of the extracted specimens, the perilimbal sclera stress-strain and relaxation tests give the properties of the tissue in the circumferential direction around the cornea.

Specimens were similarly extracted using the same dog-bone cutting die from the posterior sclera and cornea. For the posterior sclera, specimens were extracted superior and inferior to the optic nerve head, 4 mm from the center of the nerve, with the axis of the die in the nasal-temporal direction. One sample was randomly selected for a stress-strain test and the other for a relaxation test. For the cornea, two specimens were extracted in the nasal-temporal orientation. One sample was randomly selected for a stress-strain test and the other for a relaxation test.

Stress-strain and stress relaxation tests were performed in the horizontal configuration using an actuator and controller system (TestResources Model 100-Q-225, Shakopee, MN) outfitted with a high precision load cell (TestResources Model SMT1-2.2–294, Shakopee, MN). Tests were conducted in a custom-made humidity and temperature controlled chamber fitted around the grips to maintain physiological conditions during the tests. Screw-action vice style mechanical grips were used, having serrated jaw surface to prevent sample slippage without over-tightening. The initial distance between the grips was approximately 13 mm and sample elongation was recorded to calculate engineering strain. The custom-made cutting die is a steel block having overall dimensions of 32 mm height, 29 mm cutting-surface length, and varying width sized to produce samples having 1.5 mm width in the gauge section and 4.75 mm width in the gripped ends. This uniaxial tensile method was generally used to test porcine eye rectangular samples [17–21] and dog-bone shaped samples [16]. In biomechanical testing of soft tissue specimens, there is limitation of initial slack in the specimen after its insertion in the test grip. Here tests were run from a completely unstrained state resulting in a small slack region at the start of each test where stress is zero and the grips displace. This zero-stress slack region was removed from each data curve to yield a consistent set of curves. Tensile stress-strain tests were conducted at the quasi-static strain-rate of approximately 0.005 s^-1^. Tensile stress relaxation tests involve two segments: a step-like input of strain, and hence a jump of stress, followed by a longer segment where the strain is held constant and the stress is permitted to take on whatever value necessary for the strain to be maintained constant over time. In a viscoelastic material, the stress usually reduces, or relaxes, during this phase. To conduct the tests, the machine was operated at 100 mm/min for approximately 1.7 s causing specimens to strain at the peak rate of approximately 0.13 s^-1^ until the measured stress reached 2 MPa. Strain values of perilimbal sclera specimens at 2 MPa ranged from 0.11 to 0.2. The actuator was programmed to then maintain constant specimen displacement and to measure the load as a function of time for approximately four minutes as it relaxes.

To validate the mechanical test methods and results, comparison of the present stress-strain measurements was made against stress-strain values extracted from tensile test data for porcine sclera in the published literature [17, 18].

### Viscoelastic model

**Fig 2:**
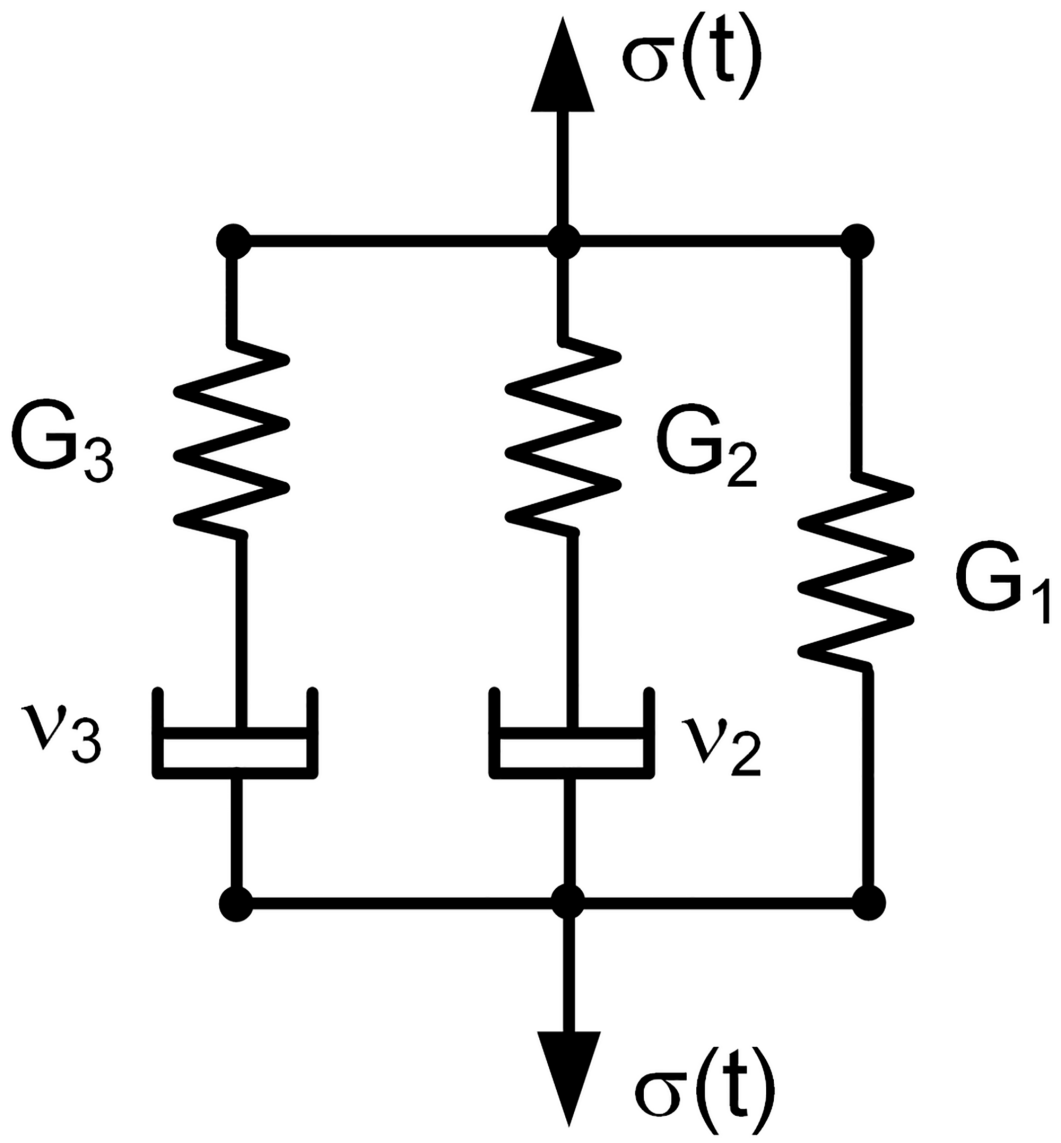
Five-parameter viscoelastic model having 3 branches. Two Maxwell branches each containing a spring and damper in series, and a third branch consisting of a single spring.

The stress relaxation curves were fit using a Maxwell-type standard linear solid viscoelastic constitutive model [22, 23] with five parameters consisting of two pairs of linear spring-damper series Maxwell units that are parallel to each other and also to a third spring, G_1_ (Fig 2). The Maxwell branches accommodate the distinct viscoelastic properties of the two primary tissue phases involving the collagen fibrils and proteoglycan matrix, while the spring G_1_ prevents unconstrained flow of the overall viscoelastic composite. Methods to determine governing constitutive equations from mechanical analog models are described elsewhere [24]. The governing equation for the present model is:

$$\begin{array}{l} \frac{1}{G_{1}G_{2}G_{3}}\frac{d^{2}\sigma (t)}{dt^{2}}+\left(\frac{1}{G_{1}G_{2}\nu_{3}}+\frac{1}{G_{1}\nu_{2}G_{3}}\right)\frac{d\sigma (t)}{dt}+\frac{1}{G_{1}\nu_{2}\nu_{3}}\sigma (t)=\\ \left(\frac{1}{G_{1}G_{2}}+\frac{1}{G_{2}G_{3}}+\frac{1}{G_{3}G_{1}}\right)\frac{d^{2}\varepsilon (t)}{dt^{2}}+\left(\frac{1}{G_{1}\nu_{2}}+\frac{1}{G_{1}\nu_{3}}+\frac{1}{G_{2}\nu_{3}}+\frac{1}{G_{3}\nu_{2}}\right)\frac{d\varepsilon (t)}{dt}+\frac{1}{\nu_{2}\nu_{3}}\varepsilon (t) \end{array}$$ (2)

Here, G_2_ and G_3_ are the elastic moduli of the springs in the Maxwell branches. G_1_ is the elastic modulus of the third spring. ν_2_ and ν_3_ are viscosity constants of the dampers in the Maxwell branches.

This model was used with measured relaxation data to determine viscoelastic constants for the eye tissue. To determine the relaxation function, Eq (2) was solved for the case in which a step function of strain of magnitude ε_o_ is applied to the model, resulting in:

$$R(t)=\varepsilon_{o}\left(G_{1}+G_{2}e^{-\frac{t}{\tau_{2}}}+G_{3}e^{-\frac{t}{\tau_{3}}}\right)$$ (3)

Here, R(t) is the stress during the relaxation phase of the response, ε_o_ is the step input of strain, and τ_2_ = ν_2_/G_2_ and τ_3_ = ν_3_/G_3_. The parameters τ_2_ and τ_3_ are time constants that result from the two Maxwell branches of the model and offer flexibility for the system to capture two distinct relaxation times as is expected to occur during relaxation of the eye tissues. Eq (3) was fit to the measured stress relaxation data using nonlinear least squares with the Levenberg-Marquardt algorithm [25].

The stresses, σ_1_, σ_2_ and σ_3_, that occur in the three branches of the model as the total stress R(t) relaxes are given by:

$$\sigma_{1}(t)=\varepsilon_{o}G_{1},\;\sigma_{2}(t)=\varepsilon_{o}G_{2}e^{-\frac{t}{\tau_{2}}},\;\sigma_{3}(t)=\varepsilon_{o}G_{3}e^{-\frac{t}{\tau_{3}}}$$ (4)

The relations show that the first branch, which lacks a damper, does not contribute to the relaxation, while the other two branches have distinct relaxation time parameters.

### Statistical and computational methods

The primary outcomes of interest were the association of IOP and net outflow facility with tissue biomechanical properties. Descriptive statistics (mean and standard deviation) are reported for IOP and net outflow facility at the two perfusion rates.

Statistical computations, graphics, and numerical calculations were conducted in Maple (version 17.01, Waterloo Maple Inc., Waterloo, ON), Matlab (Release 2014 The MathWorks, Inc., Natick, MA), and JMP^®^ Pro Version 13 (SAS Institute Inc., Cary, NC, 1989–2007). Distributional assumptions of normality were checked graphically with normal quantile plots (q-q plots) [26], and quantitatively with Shapiro-Wilk W goodness of fit tests [27].

On scatter plots, the bivariate 95% confidence ellipses are displayed. The ellipses indicate where 95% of the data are expected to lie given a bivariate normal distribution. As a graphical indicator, the ellipses reflect the correlation between the two displayed variables, with a more circular shape indicating less correlation [28]. Also shown on the scatter plots are regression lines resulting from ordinary least squares regression to indicate the y-axis variable trend of each group over the range of the x-axis variable. Coefficients of determination are shown as R^2^.

## Results

### IOP and net outflow facility results

Eleven porcine eyes from eleven different animals were successfully tested. Supplementary figure S1 Fig shows a typical trace of the IOP vs. time behavior. The preconditioning phase followed by convergence to steady pressure at each flow rate are indicated on the trace. Results of the outcome measures are given in Table 1 with the steady IOPs at the two flow rates (P_1_ at 3 μl/min, P_2_ at 6 μl/min), the net outflow facility calculated from both pressures and the flow rates, and the tangent modulus discussed later. The average outflow facility at each flow rate increases from 0.43 (μl/min)/mmHg at 3 μl/min to 0.64 (μl/min)/mmHg at 6 μl/min. These outflow facility values are within the published results of porcine eyes, 0.26–1.09 (μl/min)/mmHg [29–33], varying significantly depending on test conditions.

**Table 1:** Whole globe perfusion and perilimbal sclera specimen stress-strain data.

Eye Number	IOP P_1_ (mmHg) at 3 μl/min	IOP P_2_ (mmHg) at 6 μl/min	Net Outflow Facility (μl/min)/mmHg)	Tangent Modulus (MPa)
1	10.0	10.9	3.33	6.1
2	8.3	9.5	2.50	5.7
3	9.3	14.6	0.57	6.9
4	6.5	8.4	1.58	4.8
5	5.9	8.7	1.07	5.5
6	9.0	11.2	1.36	7.1
7	12.5	14.5	1.50	8.9
8	6.8	10.8	0.75	5.8
9	3.3	5.0	1.76	4.7
10	7.5	9.0	2.00	5.1
11	7.4	8.5	2.73	6.1
Mean	7.86	10.10	1.74	6.07
SD	2.40	2.77	0.85	1.21

### Biomechanical test results

**Fig 3:**
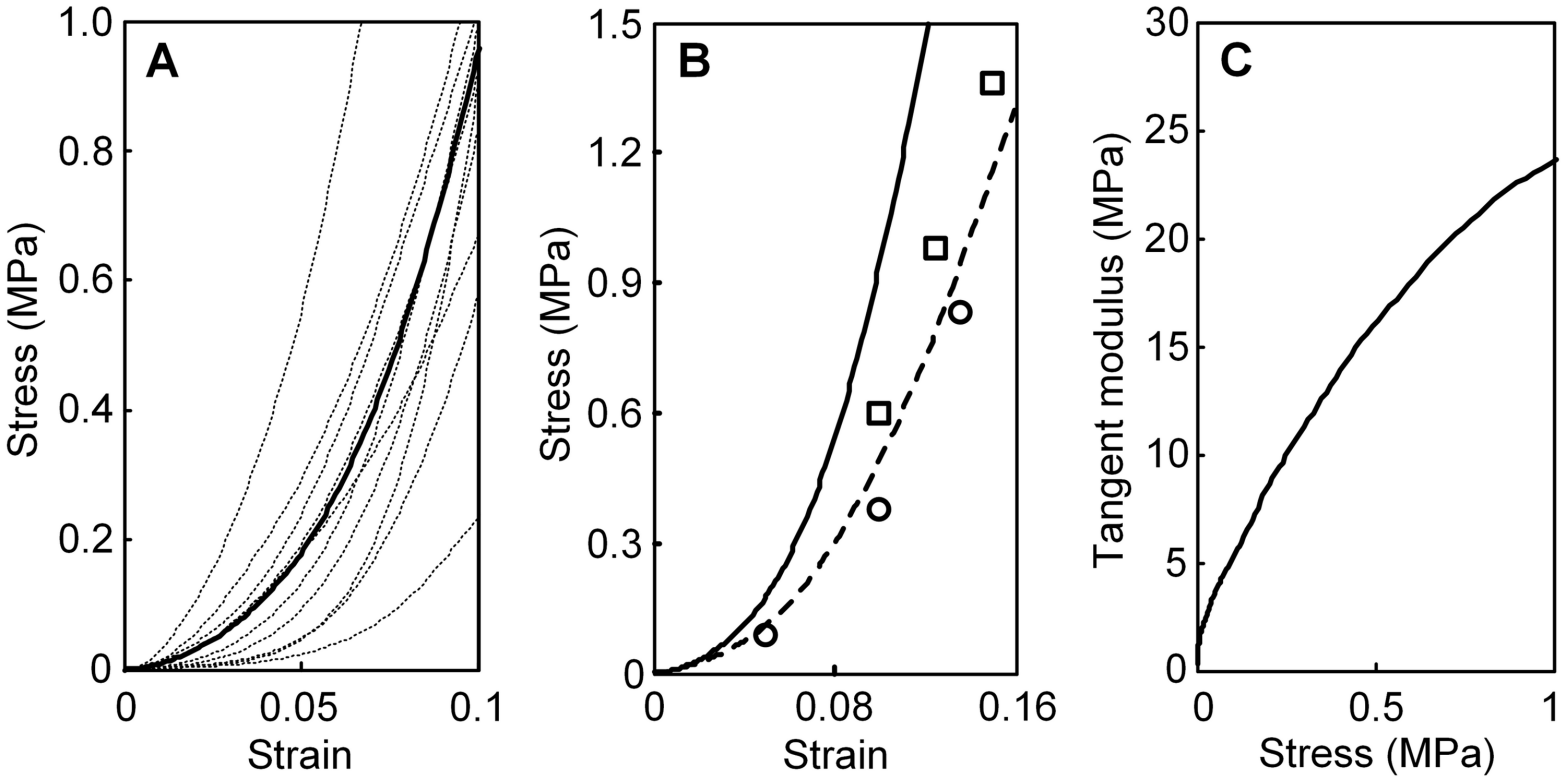
Results of mechanical stress-strain tests on perilimbal and posterior sclera samples at the strain rate of 0.005 s^-1^. (A) Raw stress-strain data (thin dotted lines) and average (thick solid line) for the perilimbal samples. (B) Average stress-strain curves of perilimbal sclera (solid line) and posterior sclera (dashed line). Circle [18] and square [17] marker points are from previously published data for posterior sclera. (C) Tangent modulus versus stress extracted from the average curve in Fig 3A.

The average stress-strain curve is shown in Fig 3A, for ten perilimbal sclera samples along with the individual raw data curves tested at the quasi-static strain-rate of 0.005 s^-1^. One of the 11 samples slipped in the testing grips at very high strain and so has been excluded from the curve averaging. The stress-strain test methods and results were validated against accepted, published results for posterior sclera in Fig 3 of Wollensak et al. [18] and Fig 4 of Spoerl et al. [17]. Other results focused on porcine perilimbal sclera are not available, to the authors’ knowledge. The comparison is given in Fig 3B in which circle and square marker points were determined from [18] and [17], respectively, and the dashed line denotes the average stress-strain curve of 11 posterior sclera samples. The present results and those in the references [17, 18] are comparable. The average stress-strain curve (solid line) of perilimbal sclera is also presented in Fig 3B for comparison.

In Fig 3C, the average tangent modulus, extracted from the average stress-strain curve of Fig 3A, is plotted versus stress. Tangent modulus is a measure of the tissue’s stiffness. The J-shaped nonlinearity of the stress-strain curve shows that the tangent modulus increases as stress increases, as seen in Fig 3C and observed in other soft tissue specimen tests. Tangent moduli values at the stress of 0.1 MPa are summarized in Table 1 for the 11 perilimbal sclera specimens. The stress of 0.1 MPa corresponds to strain of about 3.8% in the perilimbal sclera which is a realistic.

**Fig 4:**
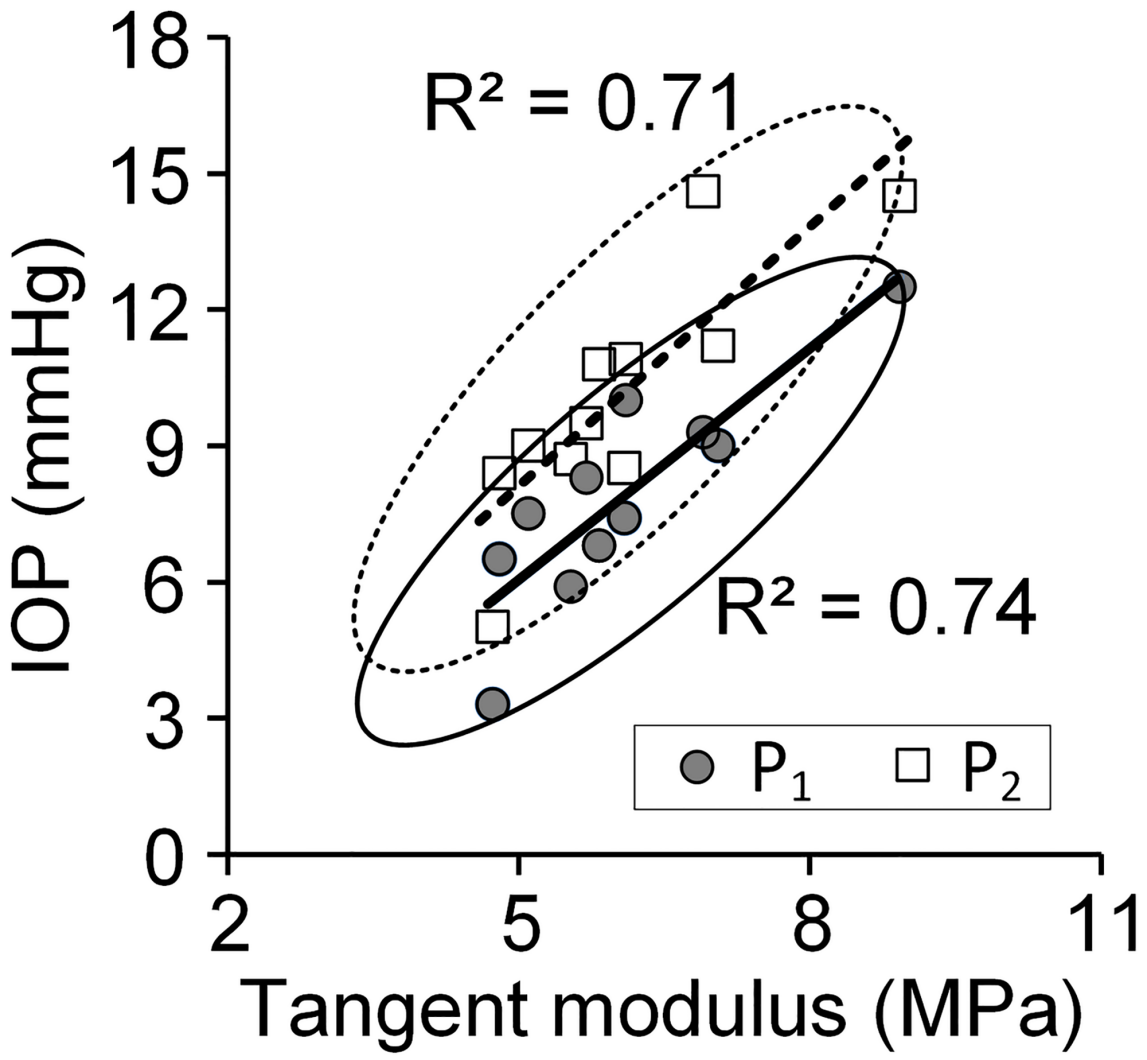
Intraocular pressures P_1_ (shaded circles) and P_2_ (squares) of eyes measured during perfusion vs. tangent modulus of perilimbal tissues dissected from the same eyes. For P_1_, R^2^ and p- values are 0.74 and 0.0006, respectively. For P_2_, R^2^ and p- values are 0.71 and 0.0011, respectively.

In Fig 4, the IOPs (P_1_ and P_2_) measured during perfusion of the eyes at the two flow rates u_1_ and u_2_ are plotted against the tangent moduli measured for perilimbal specimens from the same eyes. The bivariate confidence ellipses show the 95% confidence interval around the mean for the group at each IOP. Regression lines and R^2^ values indicate that the variation in IOP is increased by taking into account the tangent modulus. Specifically, there is a strong tendency for the IOP to increase with tangent modulus of the perilimbal sclera. A comparison was also made of IOP versus tangent moduli of the posterior sclera and cornea specimens from the same eyes (see S2 Fig). Results are summarized in Table 2 and indicate weak correlation between the eyes’ IOP and their posterior sclera and cornea tangent moduli.

**Table 2:** R^2^ values indicating degree of correlation between IOPs and tangent moduli of eye tissues.

	Perilimbal sclera	Posterior sclera	Cornea
At P_1_	0.74	0.17	0.02
At P_2_	0.71	0.30	<0.01

**Fig 5:**
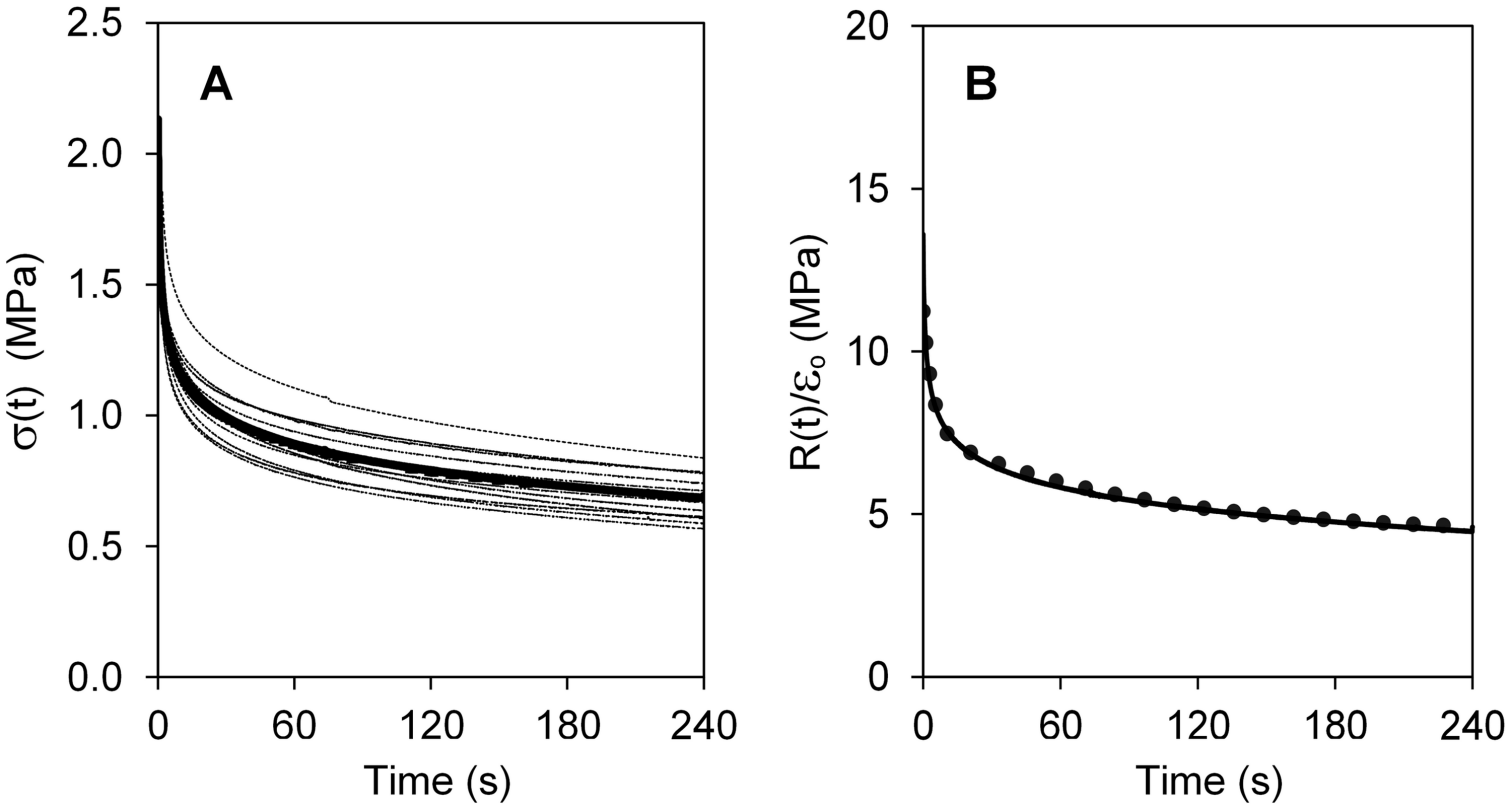
Relaxation test curves. (A) Raw stress-relaxation data of samples from 11 perfused eyes (dotted lines) and average (solid line); (B) Average relaxation curve (solid line) and five-parameter standard linear solid viscoelastic curve fit using the average viscoelastic properties (points).

In Fig 5A, the perilimbal sclera relaxation test curves and their average curve are shown. For each case, the initial, nearly vertical line is the sharp increase in stress produced by movement of the test machine grip at 100 mm/min for approximately 1.7 s. The subsequent curve is the tissue force relaxation that occurs while the displacement of the grips is held constant. This part of the data curve was fit by the five-parameter model given in Eq (3). The curve-fit given by Eq (3) matched the test data well throughout the entire relaxation time range for all specimens. To confirm that the model replicates the data, Fig 5B shows, with a solid line, the average test curve, and with dots, the calculated values from Eq (3) using the average properties. The goodness of fit of the calculated values to the measured data is R^2^ = 0.98.

**Fig 6:**
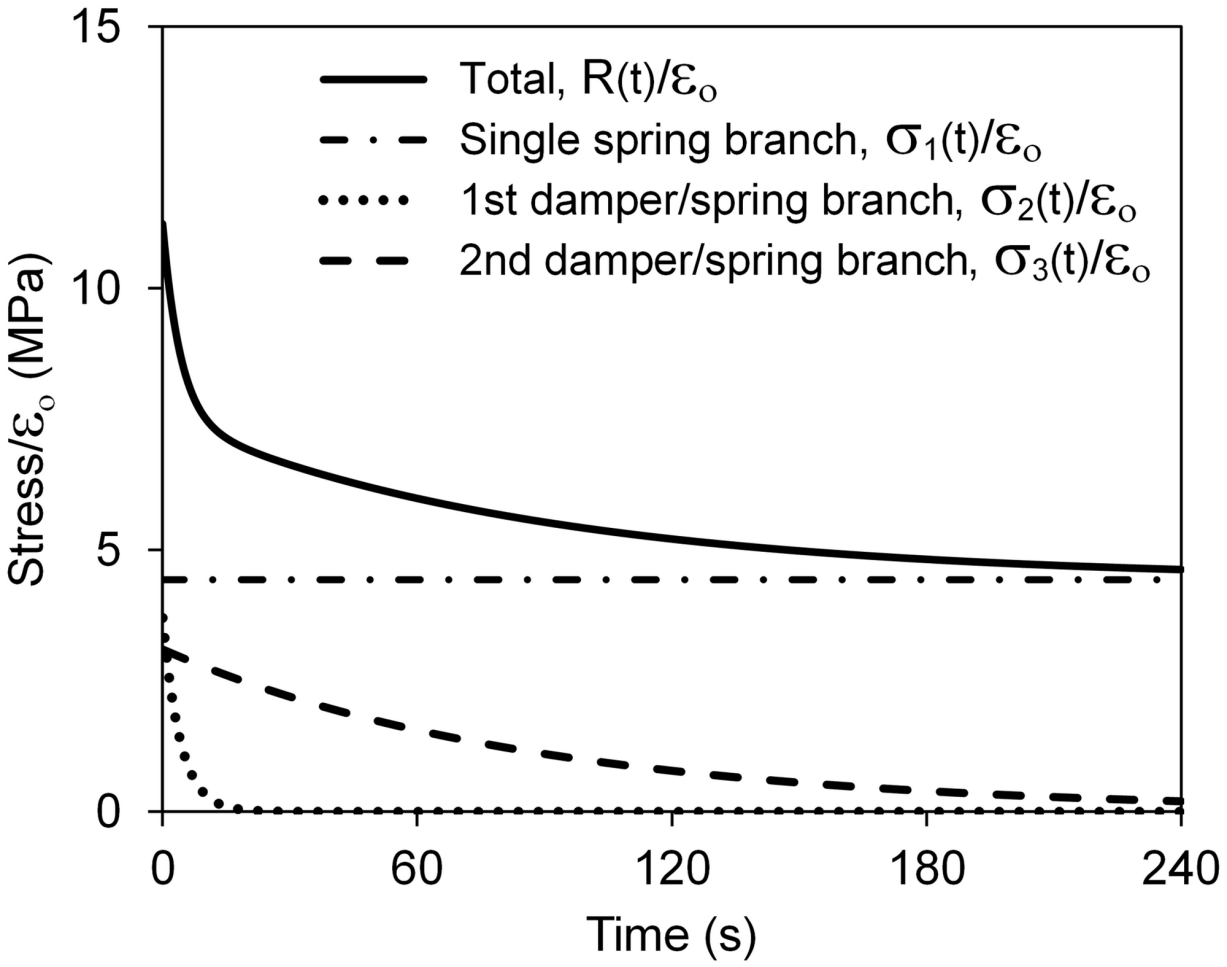
Total stress and stresses in the branches of the 5-parameter solid model calculated using Eqs (3) and (4) with the average viscoelastic properties of the eyes.

The perilimbal sclera’s average viscoelastic constants are given in Table 3. The relaxation times τ_2_ and τ_3_ corresponding to the two Maxwell branches are widely spaced indicating that the tissue stress relaxes at two distinct time phases. This can be seen in Fig 6 which shows the relaxing stresses σ_1_, σ_2_, and σ_3_ from Eq (4) divided by strain in the three branches of the model (Fig 2) along with the total stress, R(t), from Eq (3). The figure shows how the total stress in the tissue composite at any time is the sum of the stresses in the three branches of the model.

**Table 3:** Viscoelastic properties of perilimbal sclera specimens.

G_1_ (MPa)	G_2_ (MPa)	G_3_ (MPa)	ν_2_ (MPa^.^s)	ν_3_ (MPa^.^s)	τ_2_ (s)	τ_3_ (s)
4.4 ± 1.0	3.7 ± 0.9	3.1 ± 0.8	15.4 ± 4.2	269.6 ± 92.4	4.2 ± 1.2	86.0 ± 14.3

**Fig 7:**
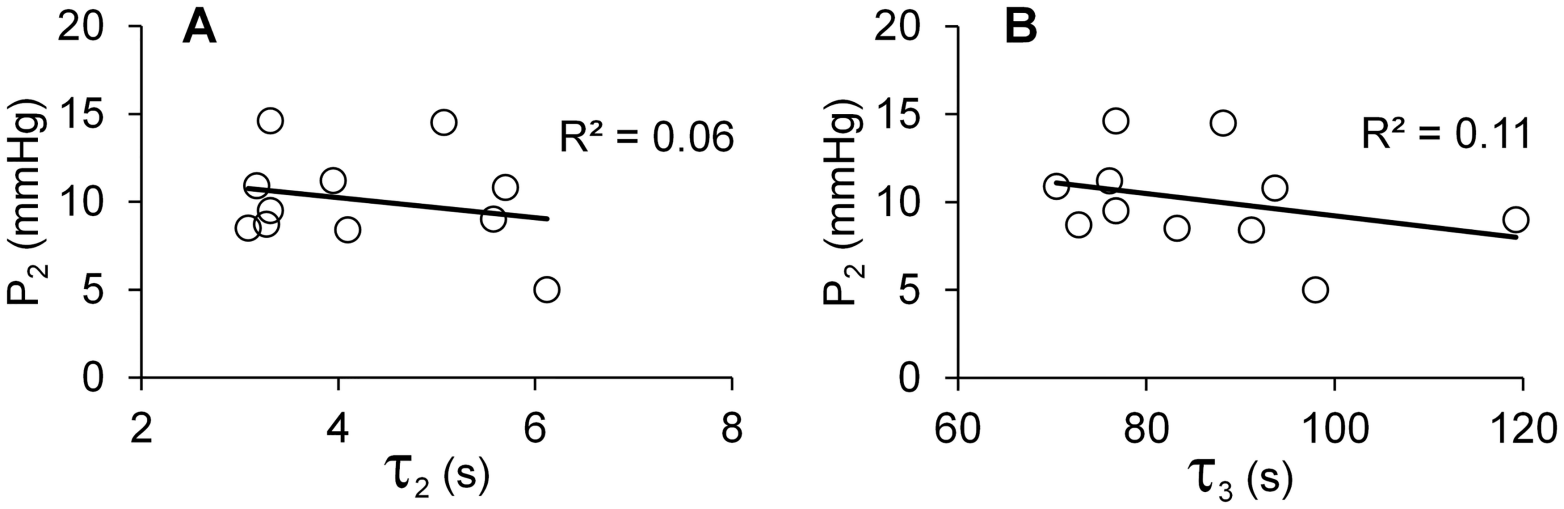
Intraocular pressures P_2_ of eyes measured during perfusion vs. relaxation times, (A) τ_2_ and (B) τ_3_, of perilimbal tissues dissected from the same eyes.

In Fig 7, the IOP (P_2_) measured during perfusion of the eyes at the flow rate of 6 μl/min is plotted against the viscoelastic constants τ_2_ and τ_3_ measured for perilimbal specimens from the same eyes. Similar plots of P_1_ versus τ_2_ and τ_3_ are presented in S3 Fig. In contrast to the tangent modulus results given in Fig 4, a correlation between IOP and τ_2_ and τ_3_ did not occur which implies that tissue viscoelasticity is not a strong variable that explains the IOP changes observed in this study.

## Discussion

Our purpose was to assess the relationship between perilimbal scleral properties and the eye’s mechano-biological response during perfusion as gauged by IOP and the calculated net outflow facility. Fig 4 shows a novel observation of a strong association between higher IOP in eyes with perilimbal sclera having greater tangent modulus. Table 2 shows that a strong association did not occur between IOP and the tangent modulus of the cornea and posterior sclera. Calculations were also made relating the IOPs to the viscoelastic properties. Some of these results are shown in Fig 7 where IOP is seen to be independent of the relaxation times. Similarly, a trend did not appear when both IOPs were plotted against the perilimbal sclera’s other viscoelastic parameters G_1_, G_2_, G_3_, ν_2_ and ν_3_ (R^2^ ≤ 0.08 in all cases) and when net outflow facility was plotted against the perilimbal sclera’s tangent modulus (see S4 Fig) and viscoelastic parameters (R^2^ ≤ 0.12 in all cases). Thus, these results are interpreted as evidence that perilimbal tissue stiffness contributes to IOP of porcine eyes. Elucidation of the mechanisms responsible for this effect requires further study. However, possible causes could be influences of increased perilimbal stiffness on the biomechanical function of the aqueous humor drainage pathway that includes the trabecular meshwork, Schlemm’s canal or aqueous venous plexus, collector channels, and/or aqueous veins, which reside in the perilimbal sclera.

That tangent modulus correlates with IOP and not net outflow facility C calculated by Eq (1) is explained by noting that the P_1_ and P_2_ lines are nearly parallel in Fig 4. So, the difference between P_1_ and P_2_ at low stiffness is about the same as at high stiffness. Thus C is negligibly affected by stiffness even though both P_1_ and P_2_ are affected.

The stress-strain behavior of the perilimbal sclera samples (Fig 3A) has the characteristic “J-shaped” nonlinearity typical of soft tissue [34]. The small “toe” range of low stress at the start of the curves is typical of soft tissues and is possibly attributable to initial straightening and re-orienting of collagen fibers and also effects of the proteoglycan matrix [35]. Nonlinear stiffening occurs as collagen fibers re-orient to resist the loading [36].

Mechanical test results are reported in Fig 3B for perilimbal and posterior sclera. Though results have been reported elsewhere on the regional variation of tissue properties, mechanical data focused on the perilimbal sclera has not been reported, to our knowledge. The test samples for the present perilimbal sclera tests were extracted using a very small dog-bone shaped cutting die in which the narrow gauge segment was directly within the perilimbal tissues, making these tests unique. The present posterior samples were extracted immediately adjacent to the optic nerve, similar to the samples used in the previous study [17]. The samples used in [18] were also extracted from the posterior sclera, though further anterior relative to the optic nerve compared to the samples in the present study and [17]. Fig 3B indicates the perilimbal sclera in porcine eyes is substantially stiffer than the posterior sclera adjacent to the optic nerve and further anterior.

The involuntary reduction of internal stress in a material when it is subjected to constant strain is a viscoelastic phenomenon. In this study, the relaxation times τ_2_ and τ_3_ characterize decay rates of the stress relaxation response, with larger values indicating greater viscosity apparent by longer time for the stress to reduce. A non-viscoelastic material would exhibit no relaxation (and creep), as would a tissue that lacks its characteristic viscoelasticity because of a defect, as observed in intervertebral disks [37]. Here it is seen (Fig 5B) that porcine scleral tissue relaxation is well-characterized by a five-parameter, viscoelastic solid model which allows for two distinct decay rates. The large difference between τ_2_ and τ_3_ indicates that the stress relaxes in the tissue composite as a combination of slow and fast phases that results from distinct physical mechanisms [35]. The initial drop in internal stress in the tissue is characterized by the fast relaxation constant τ_2_ and is likely primarily due to the response of collagen fibrils. The long-time reduction of stress characterized by τ_3_ likely results from shear response of the highly viscous proteoglycan matrix that binds the fibrils together [34]. Similar relaxation behavior has been measured in smooth muscle cells and attributed to mechanisms within the cell [38], periodontal ligaments [39], and the peripapillary sclera of rabbit and monkey eyes [40]. Though the viscoelastic behavior of eye tissue is important to the globe’s response to pressure and external loading, as well as its health, the governing viscoelastic parameters of the perilimbal sclera were not found in these tests to correlate strongly to outflow pressures.

These mechanical tests were uniaxial tests [17, 18, 41–45] on dissected specimens which overlooks anisotropy of the tissue and removes it from its natural physiological state. Biaxial tests [46–48], which are considerably more complex, can potentially account for some anisotropy provided the direction of the fibers in the test sample is determined before the test and the material is assumed to be orthotropic. The goal of the present study to focus specifically on the narrow band of perilimbal sclera required the use of the dog-bone shaped cutting die with a narrow gauge width (1.5 mm) and precluded a wider sample size demanded by biaxial testing. Uniaxial tests to determine general average mechanical characteristics have been widely conducted on eye specimens from the cornea [42, 49], sclera [17, 18, 41, 43], and lamina cribrosa [17]. The present tests provide properties of the perilimbal sclera in the circumferential direction around the cornea.

Alternatively, tissue strain can be characterized using globe inflation tests [50]. Strains can be compared from globe to globe to infer the relative stiffness, however, quantitative determination of the material properties of the tissue in such tests demands a complex whole globe mechanical model to relate the measured strain to the unknown tissue stresses which would not be suitable for the present study. In the present study, a rigorous, standardized method to extract consistent tissue samples was used for mechanical testing. The material property results were analyzed in the context of the net outflow facility and IOPs at two perfusion rates that occur in the eye from which the specimen was extracted. With the assumption that fiber orientations in these consistently sampled regions are similar, these tests are thought to be suitable for comparison from eye to eye and to the outflow results. Additionally, the present mechanical test results were found to be consistent with results in two separate studies [17, 18] thus validating the general mechanical test methods. Future studies will include whole globe strain imaging of the perilimbal tissues and comparison of strains to the outflow characteristics.

Other limitations of this study include a small sample size of 11 eyes. Despite this sample size, the study detected a strong correlation between IOP in eyes and tangent modulus of the perilimbal sclera from the same eyes. Furthermore, this strong correlation did not occur for posterior sclera and corneal tissues from the same eyes. Though the correlations in Table 2 between IOPs and the tangent modulus of the posterior sclera are 4.35 and 2.37 times lower than those of the perilimbal sclera, they are not as low as they are for the cornea. Thus it is possible that the posterior sclera’s stiffness weakly influences outflow, or its effect is muted by that of the perilimbal sclera which houses outflow structures and so can directly influence these structures.

In summary, these results provide new evidence that IOP in perfused porcine eyes is strongly influenced by the tangent modulus of the most anterior portion of the sclera, i.e. the limbus, and this correlation occurred in relation to IOP and not net outflow facility calculated using Eq (1). Furthermore, the viscoelastic properties of these tissues did not correlate to IOP or net outflow facility. These findings add to the growing knowledge of ocular tissue biomechanics in ophthalmology [51]. Clinical implementation to alter tissue biomechanics has been established in cornea tissue for keratoconus and post-refractive ectasia [52, 53]. Additional translational applications exist to alter the peripapillary sclera properties for glaucoma risk [17, 54]. These results will improve understanding of the relationship between the dynamic IOP in the porcine globe perfusion model with the properties of the perilimbal tissues in which the aqueous veins reside providing additional evidence on outflow regulation beyond the well-established effects of trabecular meshwork biomechanics [55, 56]. Further biomechanical testing combined with image analysis of the outflow distal structures will give clearer insight into the contribution of perilimbal scleral mechanics and IOP regulation.

## Supporting information

S1 Fig Example plot of IOP vs. perfusion time. Eye 5, P_1_ = 5.9 mmHg, P_2_ = 8.7 mmHg.
(TIF)

S2 Fig Intraocular pressures P_1_ (shaded circles) and P_2_ (squares) of eyes measured during perfusion vs. tangent modulus of tissues dissected from the same eyes: (A) posterior sclera and (B) cornea. (TIF)

S3 Fig Intraocular pressures P_1_ of eyes measured during perfusion vs. relaxation times, (A) τ_2_ and (B) τ_3_, of perilimbal tissues dissected from the same eyes. (TIF)

S4 Fig Net outflow facility of eyes measured during perfusion vs. tangent modulus of perilimbal tissues dissected from the same eyes. (TIF)
