# Supplementary figures and images for: Perilimbal sclera mechanical properties: Impact on intraocular pressure in porcine eyes
Source: PLoS One. 2018 May 2;13(5):e0195882. doi: 10.1371/journal.pone.0195882 (PMC5931674; doi:10.1371/journal.pone.0195882)

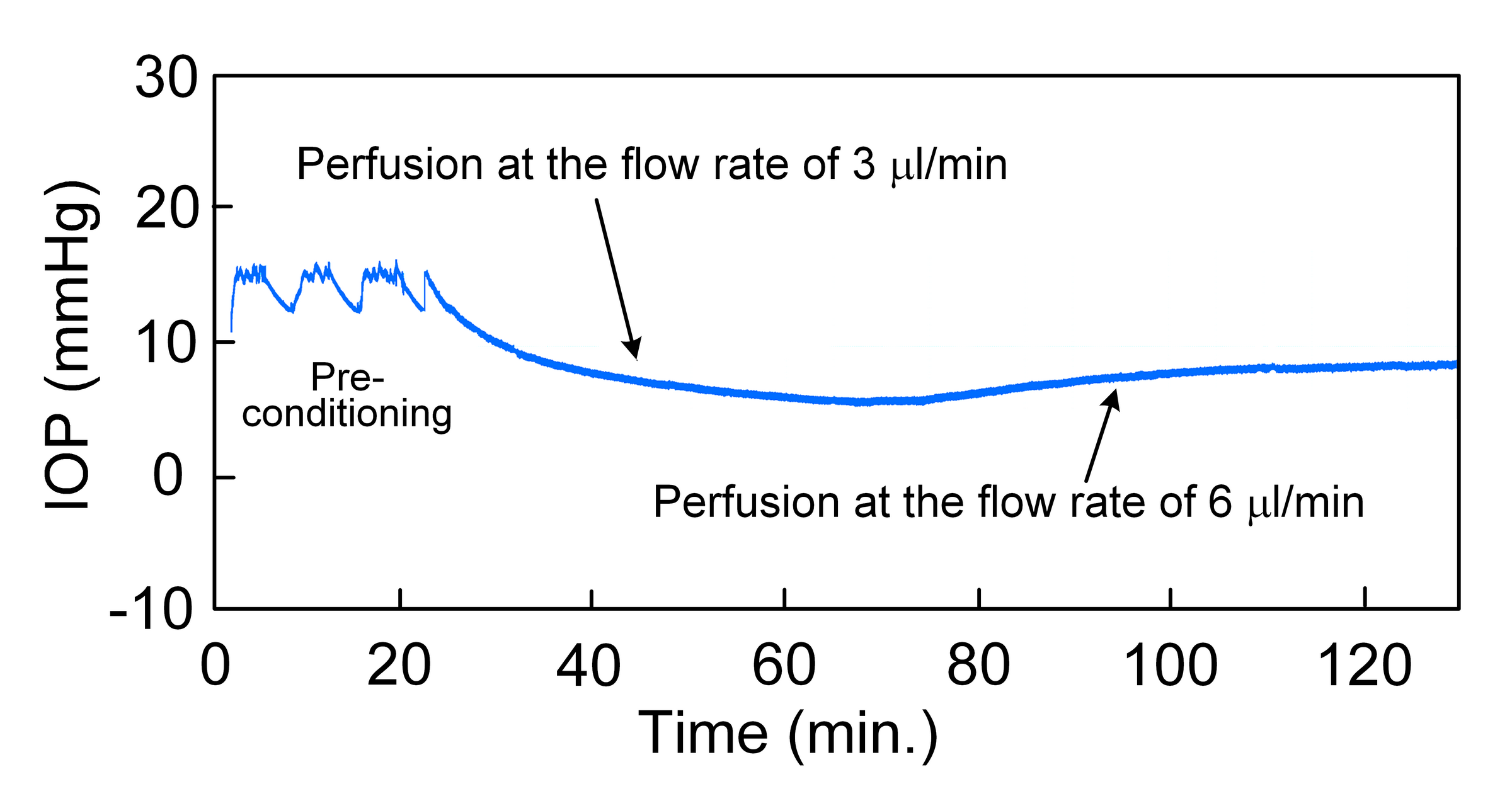

Supplement: S1 Fig — Eye 5, P1 = 5.9 mmHg, P2 = 8.7 mmHg. (TIF) [file pone.0195882.s001.tif]

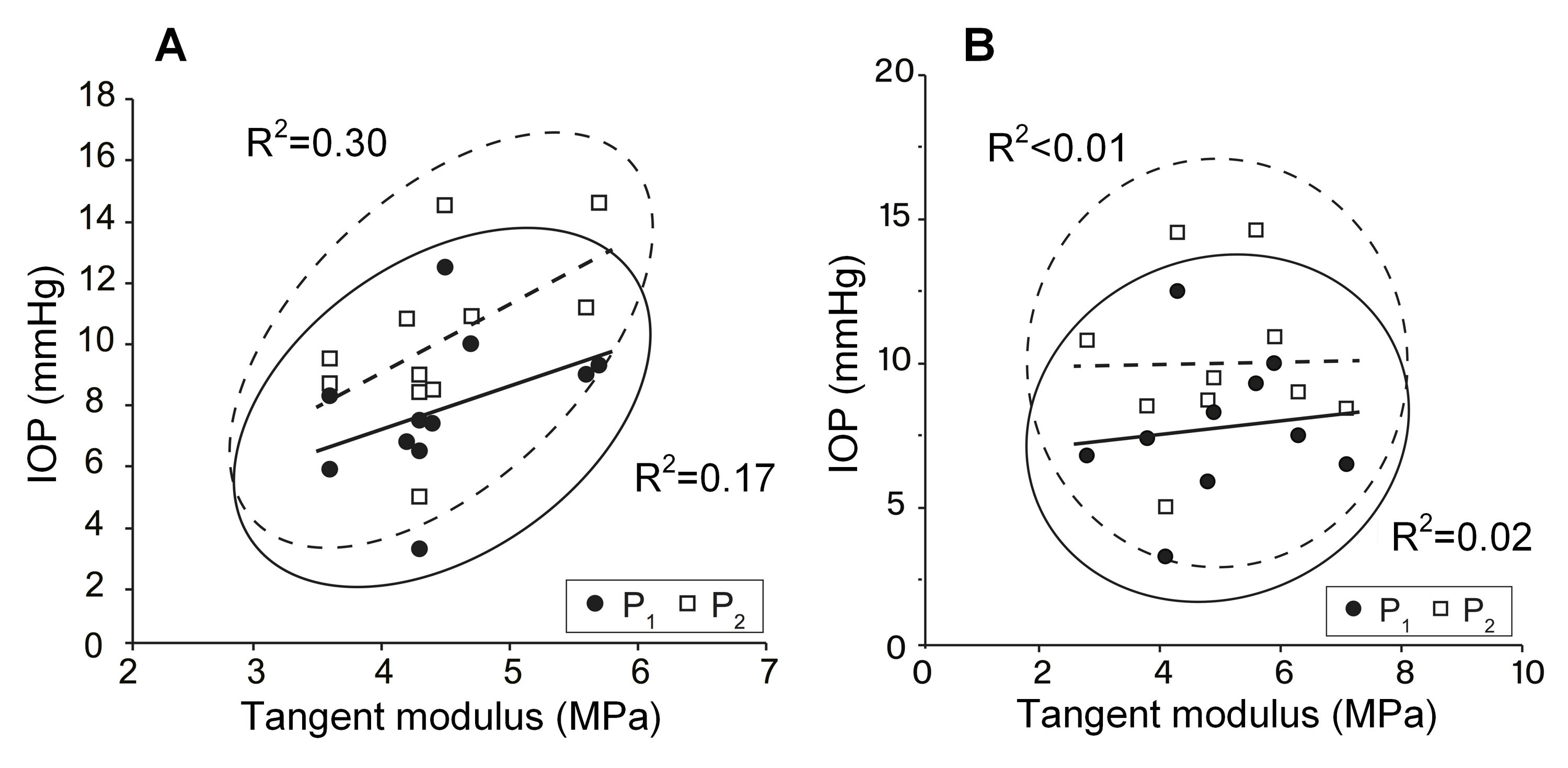

Supplement: S2 Fig — (TIF) [file pone.0195882.s002.tif]

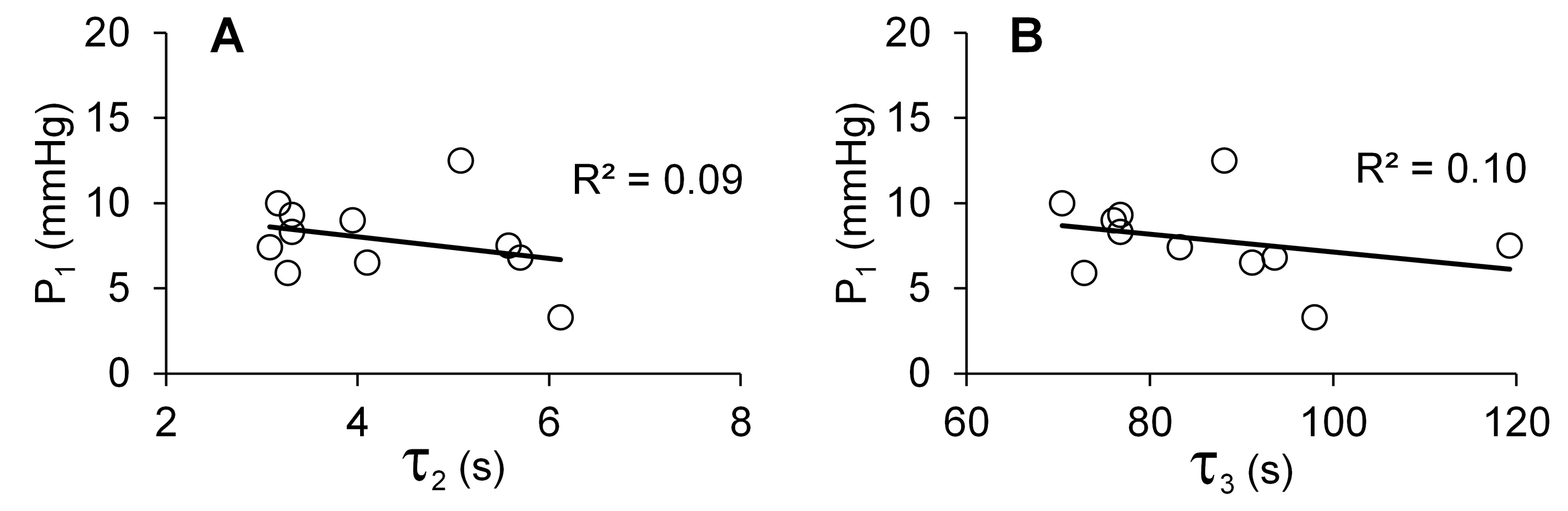

Supplement: S3 Fig — (TIF) [file pone.0195882.s003.tif]

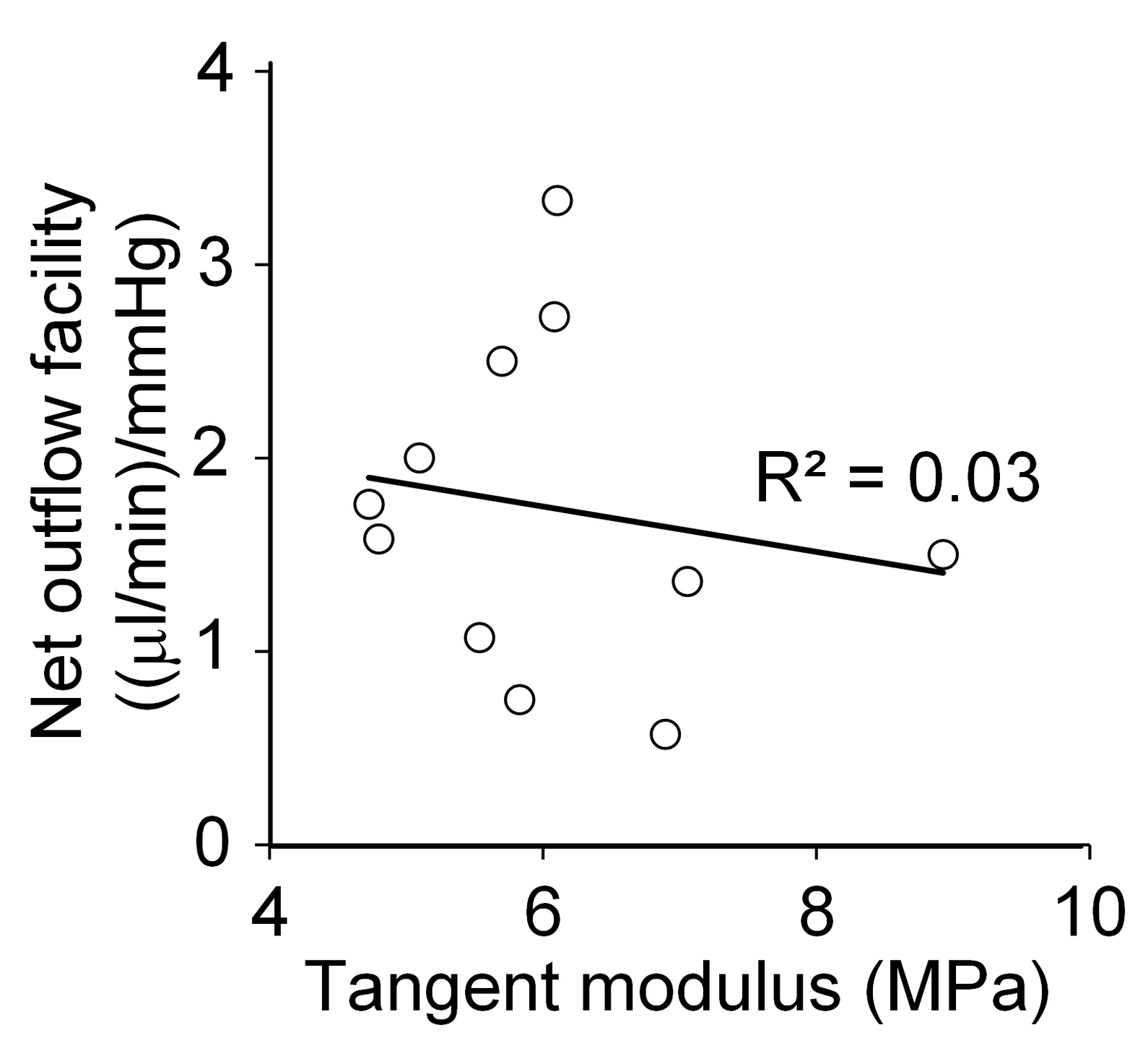

Supplement: S4 Fig — (TIF) [file pone.0195882.s004.tif]
